# Altered Percent Amplitude of Fluctuation in Healthy Subjects After 36 h Sleep Deprivation

**DOI:** 10.3389/fneur.2020.565025

**Published:** 2021-01-15

**Authors:** Bingliang Zeng, Jian Zhou, Zicong Li, Hua Zhang, Zongliang Li, Peng Yu

**Affiliations:** ^1^Department of Radiology, Jiangxi Provincial People's Hospital Affiliated to Nanchang University, Nanchang, China; ^2^Department of Imaging, The Third Affiliated Hospital of Nanchang University, Nanchang, China; ^3^Department of Imaging, The First Hospital of Nanchang, Nanchang, China; ^4^Department of Radiology, Nanfeng County People's Hospital, Fuzhou, China; ^5^Radiology Department, Jinxian County People's Hospital, Nanchang, China

**Keywords:** sleep deprivation, percent amplitude of fluctuation, receiver operating characteristic, attention network test, visual cortex, cognitive deficit

## Abstract

**Objective:** To investigate regional brain activity alteration in healthy subjects in a sleep deprivation (SD) status relative to a rested wakefulness status using a percent amplitude of fluctuation (PerAF) method.

**Methods:** A total of 20 healthy participants (12 males, 8 females; age, 22.25 ± 1.12 years) were recruited. All participants underwent attention tests and resting-state functional MRI scans during rested wakefulness before SD and after 36 h SD, respectively. The PerAF method was applied to identify SD-related regional brain activity alteration. A ROC curve was conducted to evaluate the ability of the PerAF method in distinguishing different sleep statuses. The relationships between SD-induced brain alterations and attention deficits were determined by Pearson correlation analysis.

**Results:** SD resulted in a 2.23% decrease in accuracy rate and an 8.82% increase in reaction time. SD was associated with increased PerAF differences in the bilateral visual cortex and bilateral sensorimotor cortex, and was associated with decreased PerAF differences in bilateral dorsolateral prefrontal cortex and bilateral cerebellum posterior lobe. These SD-induced brain alterations exhibited a high discriminatory power of extremely high AUC values (0.993–1) in distinguishing the two statuses. The accuracy rate positively correlated with the bilateral cerebellum posterior lobe, and bilateral dorsolateral prefrontal cortex, and negatively correlated with the bilateral sensorimotor cortex.

**Conclusions:** Acute SD could lead to an ~8% attention deficit, which was associated with regional brain activity deficits. The PerAF method might work as a potential sensitivity biomarker for identifying different sleep statuses.

## Introduction

Sleep has been increasingly shown to have far more impact on human health than it was previously recognized; however, sleep has been rarely studied by neuroimaging (1). Sleep deprivation (SD) is associated with maladaptive changes in emotion, cognition, immunity (1–9), and even expression of certain genes (10, 11). Although SD has been frequently used to explore behavioral and functional consequences caused by sleeploss (12, 13), the underlying neurobiological mechanisms of SD remain largely unknown.

During the last decade, modern brain neuroimaging techniques have been extensively used to encourage scholars to investigate the potential pernicious effects on cognitive function and regional brain areas caused by SD (4, 5, 14–18). Resting-state functional MRI (rs-fMRI) has been considered as an applicable and accepted method to address the regional brain activity deficits associated with SD. Regional homogeneity, amplitude of low-frequency fluctuations (ALFF), and fractional ALFF (fALFF) are three important methods of rs-fMRI to address regional brain alterations (5, 19–21), however, these methods could be easily influenced by physiological high-frequency respiratory and cardiac noise. A new method, namely percent amplitude of fluctuation (PerAF), has the best reliability relative to the regional homogeneity, ALFF, and degree centrality (22–24). Therefore, the proposed new method of PerAF may allow us to increase sensitivity and decrease bias when addressing the regional brain activity alternations associated with SD. However, SD has not currently been studied.

Sleep is associated with the gene transcription involved in synthesis and the maintenance of cell membrane lipids and myelin in the brain (25–27) which are particularly susceptible to insufficient sleep (27, 28). Chronic insufficient sleep and chronic stress were found to be associated with several structural changes in the brain (29, 30). Therefore, we hypothesized that SD was associated with widespread functional brain alternations, and these changes could be identified by the proposed PerAF method. To test this hypothesis, the present study utilized PerAF to identify these regional brain alternations in healthy university subjects following 36 h SD relative to a normal sleep status, which may yield insight into the neurobiological mechanisms underlying SD.

## Materials and Methods

### Subjects

A total of 20 healthy university subjects (12 males, 8 females; age, 22.25 ± 1.12 years; education, 12.8 ± 1.01 years) were recruited. All participants met the following criteria as in a previous study (1): good sleeping habits; had not used any stimulants, hypnotic medication, and psychoactive medication for at least the last 3 months; score of Pittsburgh sleep quality index lower than five, score of Hamilton Depression Rating Scale lower than seven, and score of Hamilton Anxiety Rating Scale lower than seven. The exclusion criteria met the following criteria: any history of pathological brain findings and head trauma; any foreign implants and inborn or acquired diseases; BMI >32 and BMI <19.8; any psychiatric or neurological disorders, substance dependency; and any history of sleep complaints.

The 36 h SD procedure was the same as in previous studies (1, 16), which started at 8:00 p.m. in the evening and ended at 8:00 a.m. on the third day. Our team took turns monitoring the subjects for quality control. All university subjects were required to stay awake and not allowed to sleep during the entire time of the SD procedure. All subjects were not allowed to leave the testing room and were provided with food and water during the SD procedure. All food and/or beverages did not contain caffeine, taurine, or other psychoactive substances that could influence anxiety. This study was approved by the ethical committee of our hospital. All participants were told the purpose, methods, and potential risks, and were asked to complete written informed consent.

### Attention Network Test (ANT)

All university subjects were required to conduct an attention network test (ANT) at 8:00 p.m. on the first day and at 8:00 a.m. on the third day before the MRI scans (1, 5, 31, 32). The description of the ANT was the same as in a previous study (1). The ANT comprised of three cue conditions (no cue, center cue, spatial cue) and two arrows (congruent and incongruent) in the center. Participants gave their responses by identifying the congruent or incongruent direction of central arrows. The accuracy rate and reaction time were recorded by calculating the corrected recognition and response times, respectively.

### MRI

All subjects underwent MRI scans twice, one following normal sleep and the other following 36 h SD. We used a 3.0-Tesla MR scanner (Prisma, Siemens, Germany) to finish the resting-state fMRI session. Firstly, a total of 176 slices of high-resolution anatomical volumes (repetition time = 1,900 ms, field of view= 256 × 256 mm, echo time = 2.26 ms, thickness = 0.5 mm, gap = 1 mm, flip angle = 11^0^, and acquisition matrix = 256 × 256) with sagittal orientation were acquired. Next, a total of 240 functional volumes (repetition time = 2,000 ms, acquisition matrix = 64 × 64, echo time = 30 ms, gap = 0.7 mm, thickness = 3.5 mm, flip angle = 90°, FOV = 224 × 224 mm) were collected.

### Data Analysis

Data pre-processing was performed by the RESTplus V1.2 (http://www.restfmri.net) toolbox. Firstly, the first ten functional volumes were removed. Next, other steps including form transformation, slice timing, head motion correction, spatial normalization to Montreal Neurological Institute (MNI) space and re-sampled at a resolution of 3 × 3 × 3 mm^3^, smooth (full-width Gaussian kernel = 6 × 6 × 6 mm) and linear detrend and filter (0.01–0.08 Hz) (21). Participants with more than 1.5 mm maximum translation and/or more than 1.5° degree of motion rotations in any directions were removed. Friston's 24 head motion parameters were used as covariates to regress out the effects of head motion (33–36). Linear regression was used to remove the covariates of global mean signal, white matter, head-motion, and cerebrospinal fluid signal. The PerAF method is the percentage of resting-state frequency domain of the blood oxygen level-dependent signal relative to the mean signal intensity of one given time series. After these steps of data pre-processing, the PerAF method was calculated, thus generating PerAF, mPerAF, and the z-transformation of zPerAF.

### Statistical Analysis

Two pair *t-*tests were used to calculate the ANT differences between SD status and normal sleep status by IBM SPSS 21.0. A threshold of *p* < 0.05 was considered as significant.

Firstly, one sample *t-*tests were applied to calculate the within-group differences in brain areas for sleep deprivation status and normal sleep status, separately [false discovery rate (FDR) correction, voxel-wise *p* < 0.001, and cluster-level *p* < 0.001]. Next, a two-pair *t*-test was applied to calculate between-group differences of PerAF in brain alterations [gaussian random field (GRF) correction, voxel-wise *p* < 0.001 and cluster-level *p* < 0.001, cluster voxel volumes ≥ 7,101 mm^3^]. A receiver operating characteristic (ROC) curve was frequently applied to test if neuroimaging methods might serve as potential neurobiological indicators to differentiate the two different groups (5, 16, 19, 20, 37). Here, the ROC was applied to identify the ability of the proposed PerAF method in distinguishing SD status from normal sleep status. The relationships between SD-induced brain alterations and attention deficits were determined by Pearson correlation analysis. A threshold of *p* < 0.05 was considered as significant.

## Results

### Sample Characteristics

Compared with normal sleep, acute SD showed a 2.23% poorer accuracy rate (normal sleep status, accuracy rate = 98.32 ± 1.54%; SD status, accuracy rate = 96.13 ± 2.93%; *t* = −2.961; *p* = 0.005), and an 8.82% slower reaction time (normal sleep status, reaction time = 540.5 ± 58.09 ms; SD status, reaction time = 588.16 ± 70.84 ms; *t* = 2.327; *p* = 0.025).

### PerAF Differences

**Figure 1:**
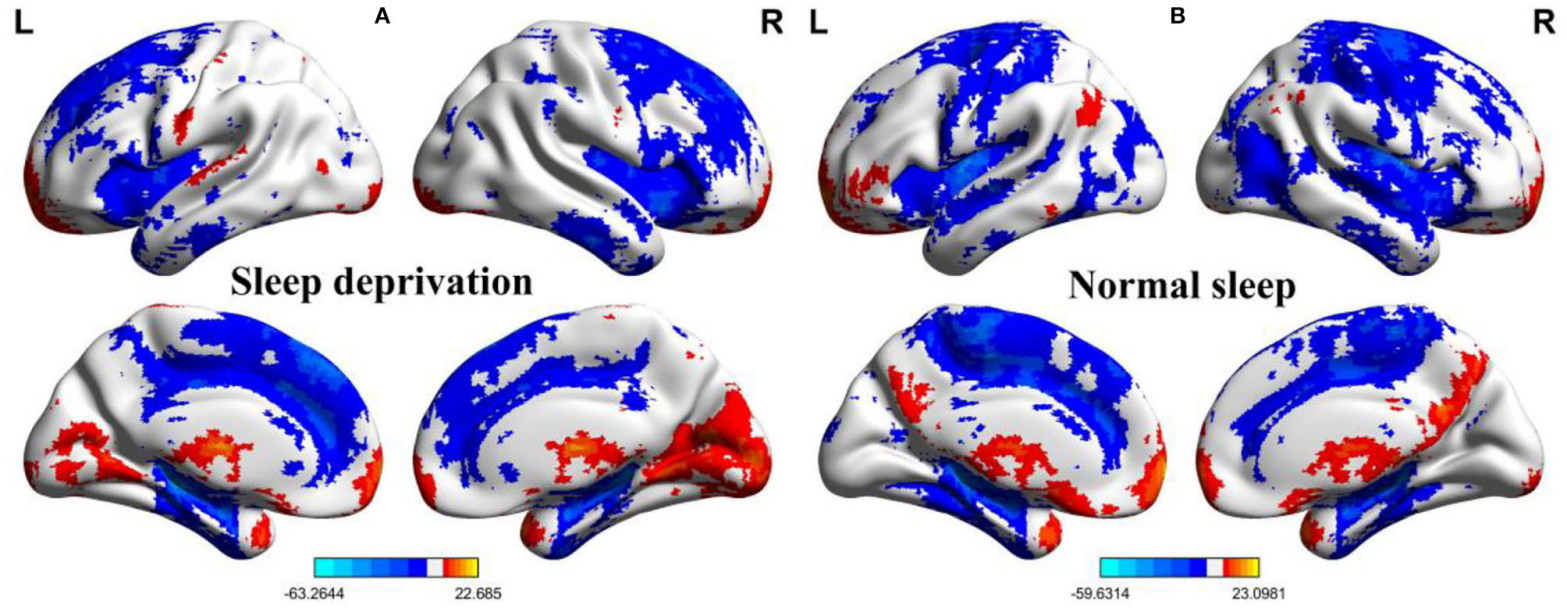
One sample *t*-test differences of SD status and normal sleep status in PerAF maps. **(A)** One sample results for sleep deprivation group; **(B)** One sample results for normal sleep group. SD, sleep deprivation; R, right; L, left; PerAF, percent amplitude of fluctuation.

**Figure 2:**
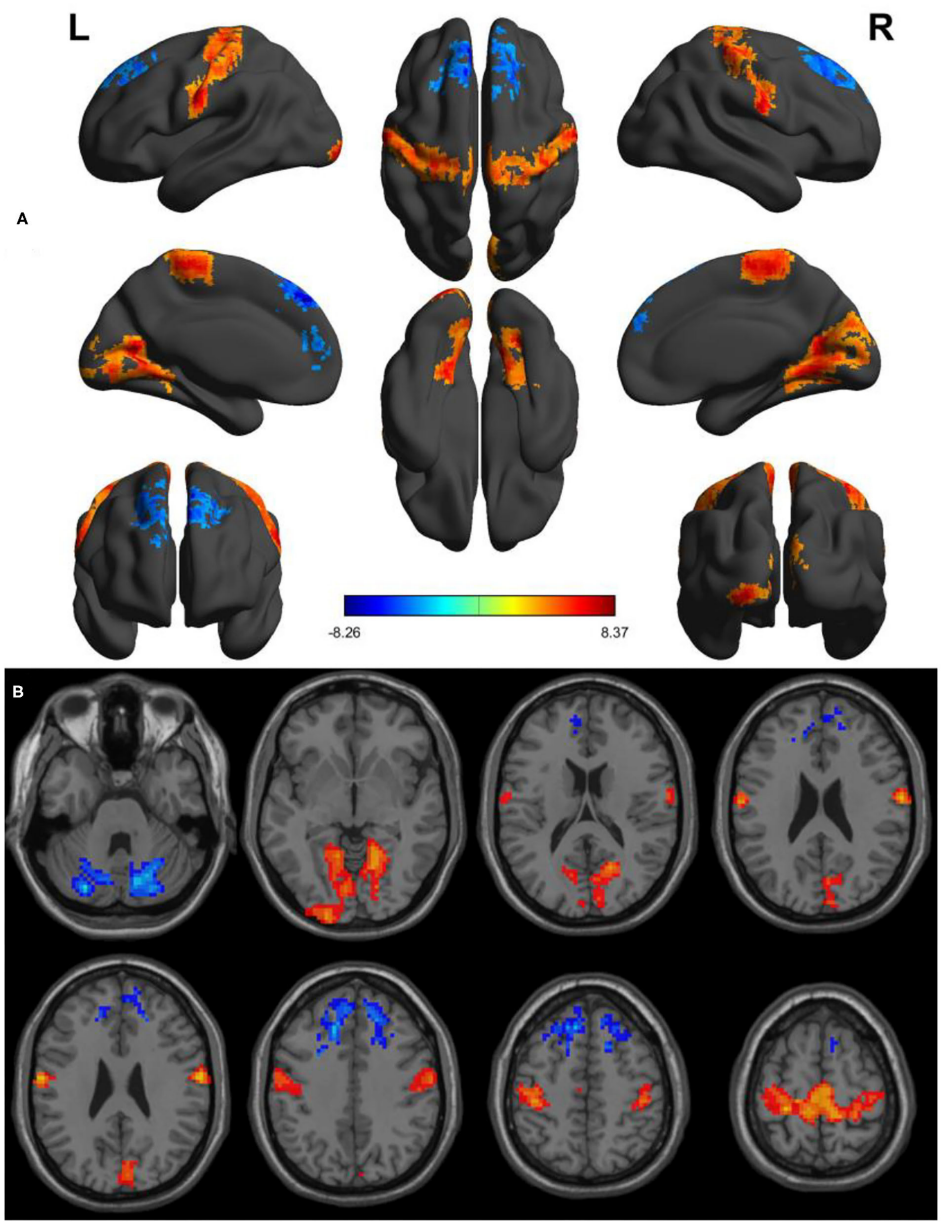
Altered PerAF between SD status and normal sleep status. **(A)** A comprehensive view; **(B)** Axial view. Red color, increased PerAF areas; Blue color, decreased PerAF areas. R, right; L, left; PerAF, percent amplitude of fluctuation; SD, sleep deprivation.

Within-group differences for the SD status (Figure 1A) and the normal sleep status (Figure 1B) are shown in Figure 1 (*p* < 0.001, FDR corrected). The within-group statistical maps showed that the covered locations of the PerAF differences in brain areas during SD status (Figure 1A) were smaller than that of during normal sleep status (Figure 1B). The between-group statistical maps showed that compared with normal sleep status, SD status showed increased PerAF differences in the bilateral visual cortex (BA 17, 18) and bilateral sensorimotor cortex (BA 3, 4), and decreased PerAF differences in the bilateral dorsolateral prefrontal cortex (BA 9) and bilateral cerebellum posterior lobe (Table 1, Figures 2A,B).

**Table 1:** The PerAF differences between SD status and normal sleep status.

**Brain regions of peak coordinates**	**R/L**	**BA**	**Voxel volume (mm^**3**^)**	**t-score of peak voxels**	**MNI coordinates**
					**X, Y, Z**
Cerebellum posterior lobe	R	N/A	916	−8.2592	36, −72, −45
Cerebellum posterior lobe	L	N/A	610	−8.1107	−30, −75, −33
Posterior cingulate, lingual gyrus, cuneus	L, R	17, 18	1,347	8.3713	−18, −66, 12
Superior frontal gyrus, medial frontal gyrus	L, R	8, 9	859	−7.6507	−6, 45, 42
Precentral gyrus, postcentral gyrus	L, R	3, 4	1,496	7.6479	15, −36, 72

*The statistical threshold was set at a corrected significance level of individual two-tailed voxel-wise p < 0.001 using a Gaussian random field corrected threshold of cluster p < 0.001 (minimum continuous cluster voxel volumes ≥ 7,101 mm^3^)*.

*PerAF, percent amplitude of fluctuation; SD, sleep deprivation; R, right; L, left; BA, Brodmann's area; MNI, Montreal neurological institute; N/A, not applicable*.

### ROC Curve

**Figure 3:**
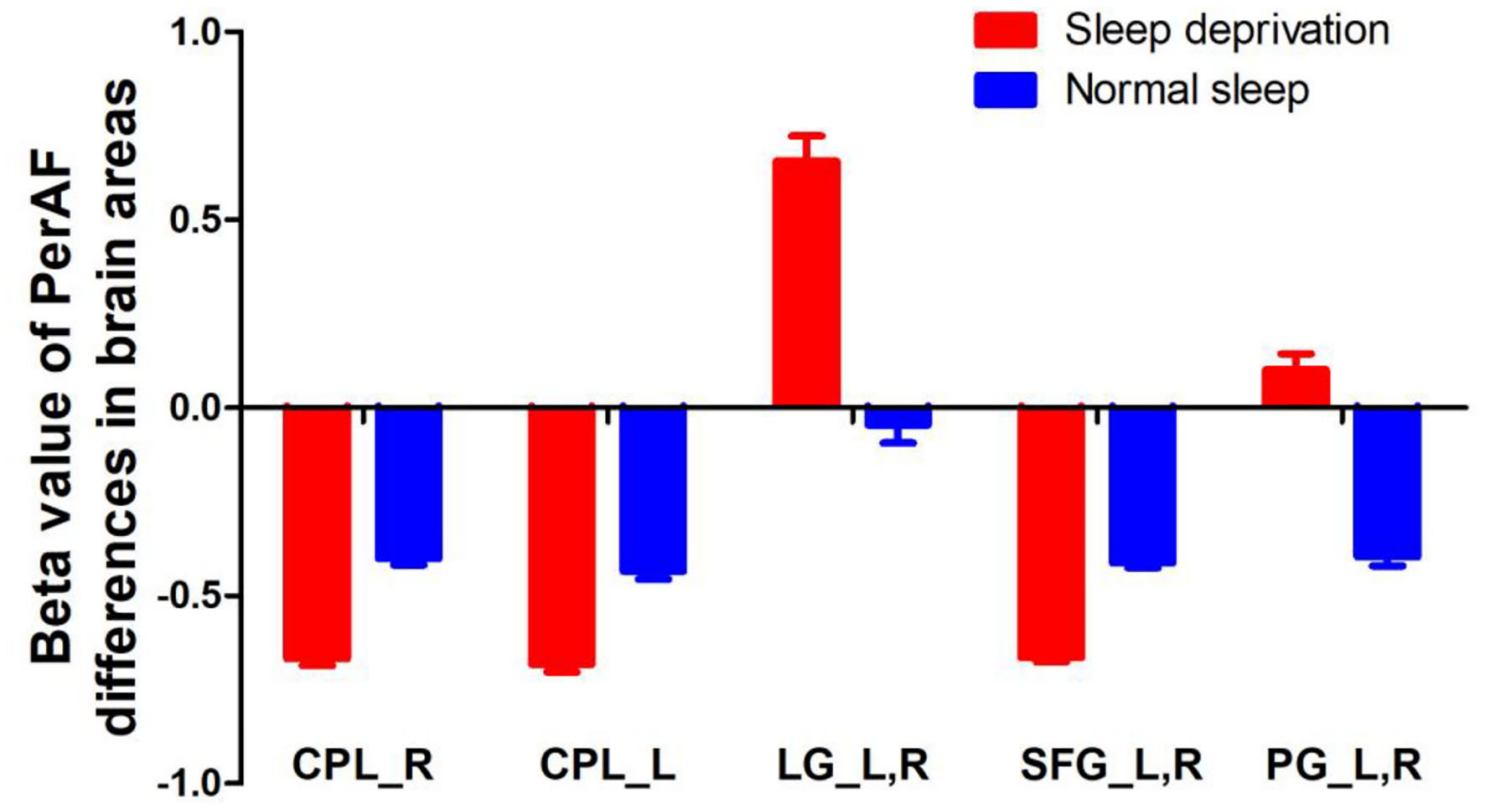
PerAF value of between-group differences in regional brain areas. PerAF, percent amplitude of fluctuation; R, right; L, left; CPL, cerebellum posterior lobe; LG, lingual gyrus; SFG, superior frontal gyrus; PG, postcentral gyrus.

**Figure 4:**
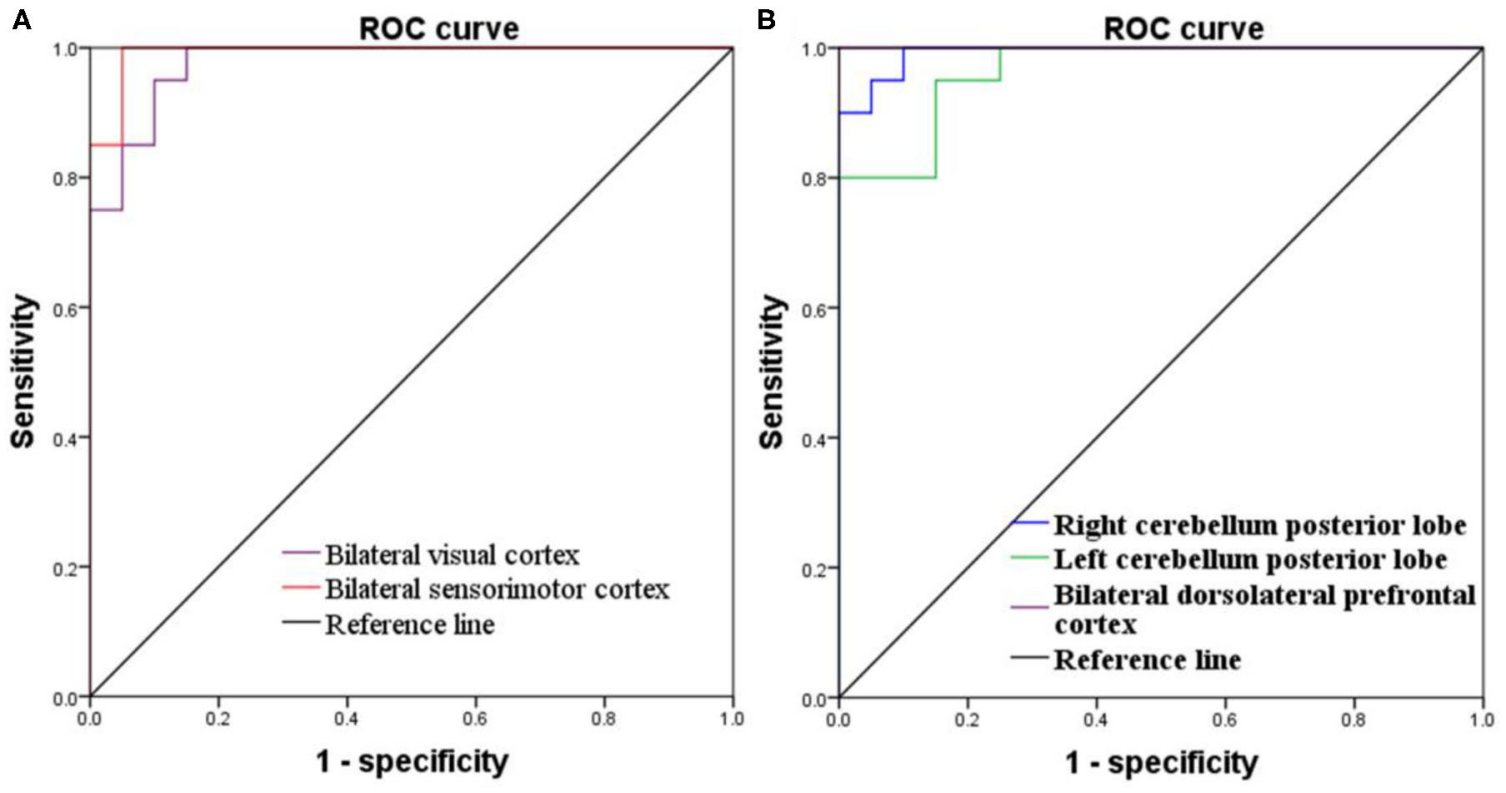
ROC curve of PerAF differences in brain areas. **(A)** Increased PerAF areas; **(B)** Decreased PerAF areas. ROC, receiver operating characteristic; PerAF, percent amplitude of fluctuation.

Since these SD-induced brain alterations exhibited differences between the SD status and normal sleep status they might serve as potential neurobiological indicators to differentiate the two different sleep statuses, we extracted the mean PerAF values of these areas for ROC curve analysis (Figure 3). Our data indicated that these areas revealed an extremely high discriminatory power with a high AUC value of 0.986 ± 0.01 (0.993–1), and further diagnostic analysis also showed a high degree of sensitivity (97 ± 2.74%, 95–100%) and specificity (93 ± 5.7%, 85–100%) (Table 2, Figures 4A,B).

**Table 2:** ROC curve for PerAF differences in brain areas between SD status and normal sleep status.

**Brain area**	**AUC, 95%Cl**	**Sensitivity, %**	**Specificity, %**	**Cut off point^a^**
R cerebellum posterior lobe	0.993 (0.976–1)	0.95	0.95	−0.5425
L cerebellum posterior lobe	0.965 (0.918–1)	0.95	0.85	−0.594
Bilateral visual cortex	0.978 (0.942–1)	0.95	0.9	0.247
Bilateral dorsolateral prefrontal cortex	1	1	1	−0.546
Bilateral sensorimotor cortex	0.993 (0.974–1)	1	0.95	−0.2215

a *Cut off point of mean PerAF signal value*.

### Pearson Correlation Analysis

**Figure 5:**
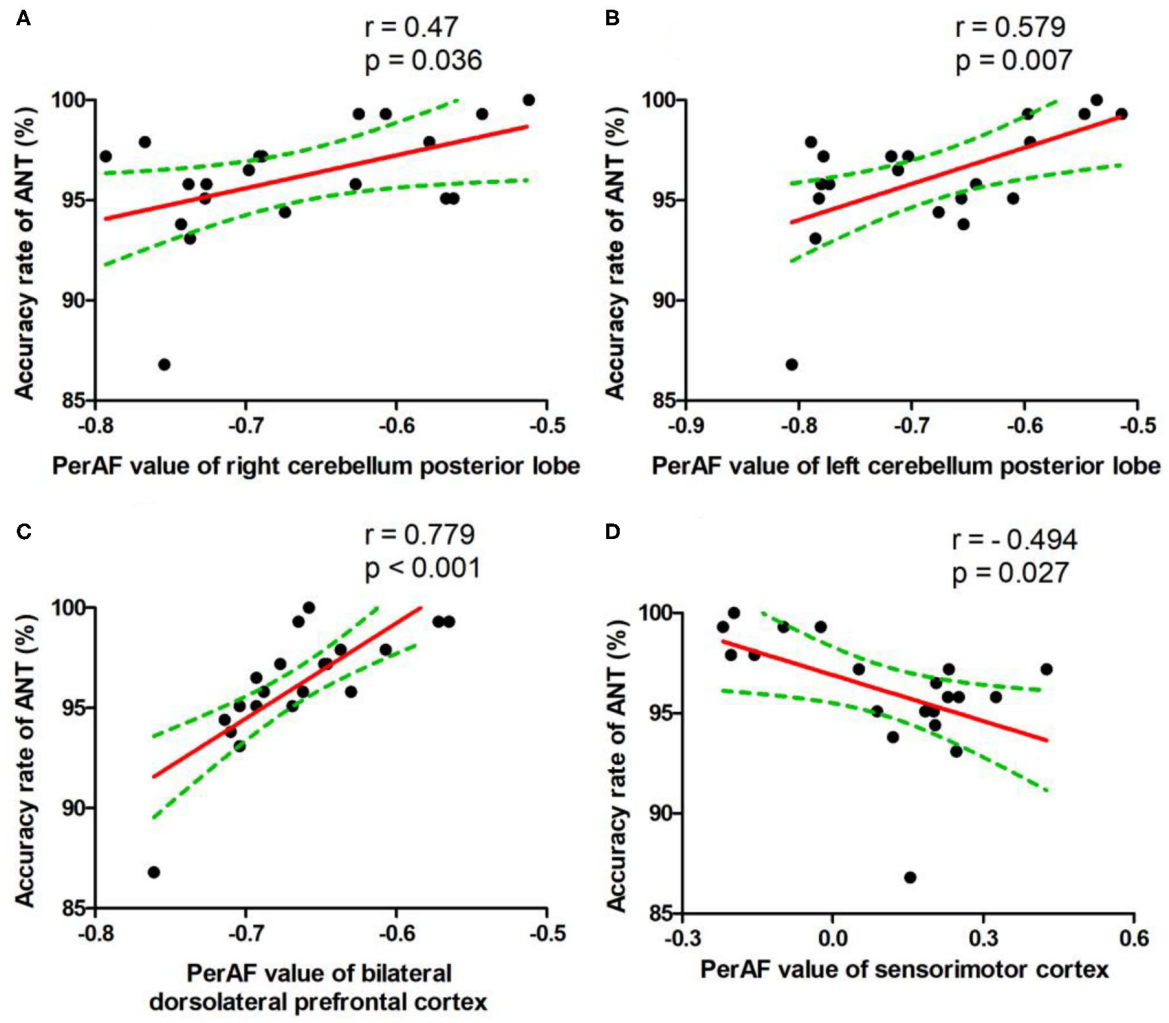
Pearson's correlation. Correlations were found between accuracy rate of ANT and bilateral cerebellum posterior lobe (Right, **A**; Left, **B**), bilateral dorsolateral prefrontal cortex **(C)**, or bilateral sensorimotor cortex **(D)**. PerAF, percent amplitude of fluctuation; ANT, attention network test.

During the SD status, several correlation analyses between the ANT differences and the SD-induced brain alterations that exhibited differences between the SD status and normal sleep status were reported (Figures 5A–D). The accuracy rate of the ANT positively correlated with the bilateral cerebellum posterior lobe (Right, *r* = 0.47, *p* = 0.036, Figure 5A; Left, *r* = 0.579, *p* = 0.007, Figure 5B) and the bilateral dorsolateral prefrontal cortex (*r* = 0.779, *p* < 0.001, Figure 5C), respectively. Furthermore, the accuracy rate of the ANT negatively correlated with the bilateral sensorimotor cortex (*r* = −0.494, *p* = 0.027, Figure 5D).

## Discussion

The present study is the first to apply the proposed PerAF method to identify SD-induced brain alterations in healthy university subjects following a total of 36 h of SD, and their relationships with the ANT. The present study reported three main findings: (a) the status transformation from normal sleep to 36 h SD in healthy subjects resulted in a 2.23% decrease in accuracy rate and an 8.82% increase in reaction time in attention; (b) SD was associated with increased PerAF differences in the bilateral visual cortex and bilateral sensorimotor cortex, and decreased PerAF differences in the bilateral dorsolateral prefrontal cortex and bilateral cerebellum posterior lobe; (c) these SD-induced brain areas exhibited an extremely high discriminatory power with extremely high AUC values (0.993–1) in distinguishing the two sleep statuses, which indicated that the PerAF method might be a potential neuroimaging indicator to differentiate different sleep statuses; (d) the accuracy rate positively correlated with the bilateral cerebellum posterior lobe and bilateral dorsolateral prefrontal cortex, and negatively correlated with the bilateral sensorimotor cortex.

Previous neuroimaging studies have shown higher regional spontaneous neural activity and short-range functional connectivity in the visual cortex in chronic insomnia patients and healthy individuals after sleep deprivation (4, 16, 18, 19, 38–41). The hyper-responses of the visual cortex has been considered as a core factor leading to the inability to initiate or maintain sleep in chronic insomnia patients (19, 39, 40, 42–46). Increased regional brain activity and functional connectivity, such as voxel-mirrored homotopic connectivity and short/long-range functional connectivity, and decreased gray matter volumes in the somatosensory cortex in chronic insomnia patients and in healthy individuals after SD status have been reported (1, 16, 37, 41). These regional brain alterations had several correlations with attention and spatial working memory deficits after the SD session (1). PET studies found that SD increased the metabolic rate of glucose in the visual and somatosensory cortex, and the metabolic rate was higher after a longer duration of SD than that of a shorter duration of SD (47, 48). Our data supported these findings. In our study, SD increased PerAF differences in the two cortexes, and these areas negatively correlated with the accuracy rate of the ANT. In this framework, we speculated that increased PerAF in these regions might be compensatory responses to the cognitive deficits. These findings support the excessive hyperarousal theory of insomnia (45).

The cerebellum posterior lobe is associated with language, cognition, and emotion, and also with the regulation of planning, initiating, and coordinating movement (4, 19, 49–51). Previous studies have shown that the cerebellum was associated with several neurologic and psychiatric diseases, including obstructive sleep apnea (52), depression (53), primary insomnia (19, 39), mood disorders (54), and sleep deprivation (4, 16, 41) and was correlated with the accuracy rate of ANT (16). Our study showed consistent findings with decreased PerAF in the cerebellum and dorsolateral prefrontal cortex, and the two areas showed positive correlations with the accuracy rate of ANT. In this framework, the decreased regional brain activity in the cerebellum and dorsolateral prefrontal cortex may suggest that the brain needs to recruit more advanced cognitive function-related brain areas to offset the attention deficits during SD, due to a continuing declined activity in these two areas during insufficient sleep status (16).

## Conclusions

In summary, the proposed method of PerAF might be a potential sensitivity neuroimaging indicator to differentiate different sleep statuses. Acute SD could lead to an ~8% attention deficit, which was associated with the increased PerAF differences in the visual cortex and sensorimotor cortex, and the decreased PerAF differences in the dorsolateral attention cortex and cerebellum. These findings could expand our knowledge of the pathophysiological mechanism of insufficient sleep and related diseases, and could provide guidance for healthcare professionals to reduce the mistakes caused by lack of sleep. However, there are several limitations that should be addressed. Firstly, the effect of differences in gender was not considered (4, 19). Secondly, the small sample sizes limited the comparisons. Thirdly, the caloric intake and sleep at baseline were not considered.

## Data Availability Statement

The raw data supporting the conclusions of this article will be made available by the authors, without undue reservation.

## Ethics Statement

The studies involving human participants were reviewed and approved by Jiangxi Provincial People's Hospital Affiliated to Nanchang University. The patients/participants provided their written informed consent to participate in this study.

## Author Contributions

BZ, ZiL, and HZ conceived and designed the whole experiment. BZ, JZ, ZoL, and PY took responsibility for the integrity of the data, the accuracy of the data analysis, and statistical data analysis. BZ wrote the main manuscript text, and under took the critical interpretation of the data. All authors contributed to the final version of the paper and have read, as well as, approved the final manuscript.

## Conflict of Interest

The authors declare that the research was conducted in the absence of any commercial or financial relationships that could be construed as a potential conflict of interest.
